# Urban - rural disparities in antenatal care utilization: a study of two cohorts of pregnant women in Vietnam

**DOI:** 10.1186/1472-6963-11-120

**Published:** 2011-05-23

**Authors:** Toan K Tran, Chuc TK Nguyen, Hinh D Nguyen, Bo Eriksson, Goran Bondjers, Karin Gottvall, Henry Ascher, Max Petzold

**Affiliations:** 1 Family Medicine Department, Hanoi Medical University, No.1 Ton That Tung Street, Hanoi, Vietnam; 2 Obstetrics and Gynaecology Department, Hanoi Medical University, No.1 Ton That Tung Street, Hanoi, Vietnam; 3 The Nordic School of Public Health, Nya Varvet Byggnad 25, Box 12133, SE-402 42 Gothenburg, Sweden; 4 Sahlgrenska Academy, University of Gothenburg, PO Box 400, SE-405 30 Gothenburg, Sweden; 5 Division of Global Health (IHCAR), Department of Public Health Sciences, Karolinska Institute, 171 77 Stockholm, Sweden

**Keywords:** Antenatal care, adequacy, disparities, urban - rural comparison, Vietnam

## Abstract

**Background:**

The use of antenatal care (ANC) varies between countries and in different settings within each country. Most previous studies of ANC in Vietnam have been cross-sectional, and conducted in rural areas before the year 2000. This study aims to compare the pattern and the adequacy of ANC used in rural and urban Vietnam following two cohorts of pregnant women.

**Methods:**

A comparative study with two cohorts comprising totally 2132 pregnant women were followed in two health and demographic surveillance sites, one rural and one urban in Hanoi province, Vietnam. The women were quarterly interviewed using a structured questionnaire until delivery. The primary information obtained was the number and the content of ANC visits.

**Results:**

Almost all women reported some use of ANC. The average number of visits was much lower in the rural setting (4.4) than in the urban (7.7). In the rural area, 77.2% of women had at least three visits and 69.1% attended ANC during the first trimester. The corresponding percentages for the urban women were 97.2% and 97.2%. Only 20.3% of the rural women compared to 81.1% of the urban women received all core ANC services. As a result, the adequate use of ANC was 5.2 times in the urban than in the rural setting (78.3% compared to 15.2%). Nearly all women received ultrasound examination during pregnancy with a mean value of 6.0 scans per woman in the urban area and 3.5 in the rural. Most rural women used ANC at commune health centres and private clinics while urban women mainly visited public hospitals. Expenditure related to ANC utilization for the urban women was 7.1 times that for the urban women.

**Conclusion:**

The women in the rural area attended ANC later, had fewer visits and received much fewer services than urban women. The large disparity in ANC adequacy between the two settings suggests special attention for the ANC programme in rural areas focusing on its content. Revision and enforcement of the national guidelines to improve the behaviour and practice of both users and providers are necessary.

## Background

Antenatal care (ANC) has been established in high income countries for a long time and has brought about remarkable achievements in reducing maternal and neonatal mortality. Most low and middle income countries have applied the same ANC programmes used in high income countries with some adjustments for local contexts [1-3]. WHO recommends a new model of ANC for women with uncomplicated pregnancy in developing countries, with at least four visits with compulsory measurement of blood pressure, testing of urine and blood tests and optional weight and height measurement at each visit [1].

The use of ANC varies between countries with a great underutilization among pregnant women in low income countries in Africa and Asia [4]. Within a country, ANC utilization also differs according to the mother's age, education, occupation, household income, parity, place of residence, cost and availability of services [5-9]. However, it is unclear whether there are rural-urban differences in ANC utilization. Some studies show that urban women use more visits and more adequate ANC than rural women while other studies report no significant difference between urban and rural areas or that urban women were significantly less likely to use ANC [10,11].

In Vietnam, pregnant women are recommended to have at least three ANC visits during the pregnancy, with one each trimester. According to the national guidelines, the ANC package includes bio-medical assessments (body weight and height, blood pressure, fundal height, fetal abdominal circumference, fetal heart rate, vaginal examination, ultrasound scan and urine and blood testing); care provisions (tetanus vaccination, iron/folate supplement) and health consultation [12]. Before the "Doi Moi" policy in 1986, pregnant women were mostly provided ANC at commune health centres (CHC) with subsidies from the government. Only women considered to have a high risk pregnancy were referred to district hospitals or higher levels. With health sector reforms including the introduction of the user fee policy and the establishment of a private health system, pregnant women now have more options for ANC even though ANC services are mostly available at primary level public health facilities. The reforms might have led to larger gaps between different regions and social groups in the use of health care in general and ANC utilization in particular [13].

In 2005, more than 80% of all pregnant women were reported to use ANC. This may have contributed to a decrease in infant mortality rate from 39 to 18 per 1,000 live births and maternal mortality ratio from 92 to 80 per 100,000 live births between 1997 and 2005 [14]. However, these mortality rates are still relatively high, especially in mountainous and rural areas. Among maternal deaths, 65% of the mothers did not use any ANC, 22% received one ANC visit and only 13% received two or more. The national target for 2010 is that 90% of women should receive at least one ANC visit during pregnancy [15].

Almost all previous studies in ANC in Vietnam have been cross-sectional, conducted before the year 2000 and in rural areas. These studies always use a single indicator of ANC utilization, mainly mentioned in quantities of ANC with the focus on number and initiation of ANC visits [16-18]. Few studies addressed the content of ANC. Studies on overall ANC adequacy using combined indicators are even rarer [19-21]. A cross sectional survey conducted at three rural provinces in the south of Vietnam in 1999 might be the first study addressing the adequacy of ANC with a combination of the number of visits, the initiation of visits and the content of ANC [21]. With the establishment of two health and demographic surveillance sites (HDSS) in rural and urban Vietnam, we have the opportunity to conduct a follow up study of pregnant women in both settings. The objective of this paper is to compare the pattern and adequacy of antenatal care used in rural and urban Vietnam. The result should lead to policy recommendations to improve maternal health care in general in Vietnam.

## Methods

### Study setting

The study was conducted in two health and demographic surveillance sites (HDSS), one in a rural district and one in an urban district of Hanoi. The purpose of the HDSS is to provide basic information for health planning and policy decisions as well as community health research and training. FilaBavi HDSS was developed in Ba Vi rural district in 1999 including 69 hamlets, selected using random sampling, with 51,024 persons in 11,089 households (20% of the district's population) [22]. DodaLab HDSS was started at the end of 2007 in 3 communes at different socioeconomic levels, selected from 21 communes of Dong Da urban district and covered about 11,000 households and 38,000 inhabitants (12% of the district's population). All inhabitants in these hamlets and communes in the two sites have been and still are under surveillance.

In the two HDSS, 106 field workers (46 in FilaBavi and 60 in DodaLab), mostly females, have been recruited and trained for data collection. They are responsible for collecting data through household interviews using structured questionnaires. The routine data collection includes quarterly follow up surveys to collect health and demographic events and a major household survey repeated every two years to update demographic and socioeconomic information at individual and household level. Almost all adult persons in the two sites are literate. In both sites, the women can receive ANC at either public or private health facilities. Filabavi has commune health centres and one district hospital, in DodaLab there are many more public hospitals and private clinics within or in the close vicinity. According to the results from the first baseline surveys in the two sites, the average distances to access the nearest public hospital are 1.8 km in DodaLab and 10.2 km in FilaBavi, based on the household GPS coordinates.

### Study design

**Figure 1:**
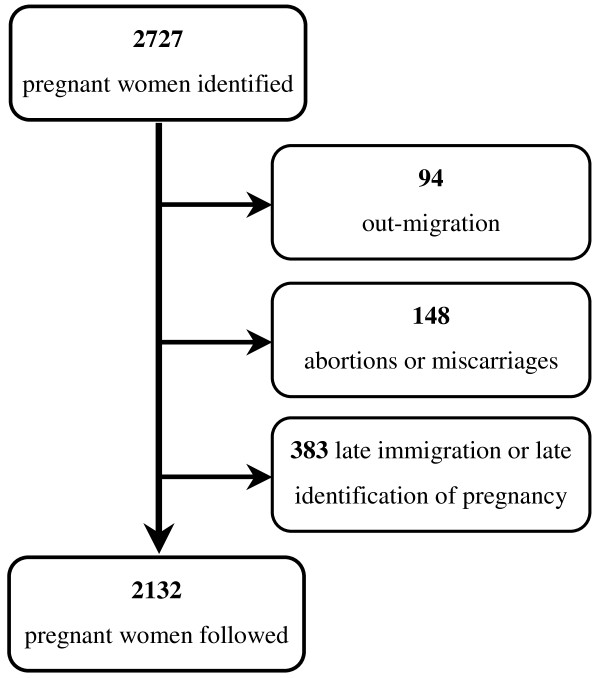
**Women identified and included in the study**.

A comparative study was conducted with two cohorts comprising all pregnant women identified through the routine surveys in the two sites from April 2008 to December 2009. The women were then followed every three months until termination of the pregnancy. A total of 2132 out of 2757 identified women were followed until delivery with at least two interviews, Figure 1.

Interviews were conducted using a structured questionnaire to obtain information about when the women made the first ANC visit, how many visits they had, where and from whom antenatal care was offered, what services were provided and how much they paid for ANC. All services in the MoH recommended ANC package were listed in the questionnaire. Data was collected by the HDSS interviewers, who were trained carefully on the content of the questionnaire as well as in general interview skills. Three percent of randomly selected forms were re-interviewed by field supervisors. For about a quarter of all women the reported information on the use of ANC was compared to local official pregnancy registration books. Acceptable agreement between the two sources of information was found. Demographic and socioeconomic information of the women (age, occupation, education level, marital status and number of children) were taken from the last major household survey in the two HDSS in 2009.

### Data analysis

Three main indicators of ANC utilization were used: (i) number of ANC visits, (ii) time for the first visit and (iii) ANC service content. ANC adequate use was defined according to the criteria of table 1 as using of enough visits (at least three) with early timing (first visit during first trimester) and sufficient services (at least six core services according to the national recommendations) [12]. High risk pregnancy was considered as women meeting any of following criteria: nullipara, age 40 or older; women with more than 4 births; women with any experiences of abortion, stillbirth, caesarean section, preterm delivery or neonatal death or women reporting any conditions such as high blood pressure, diabetes, epilepsy, depression during pregnancy [23]. Data was entered into computers with the Access application and analyzed using STATA software version 11.0. Chi square test and t-test were used for comparison of proportions and mean values respectively between the two sites. For t-test the distributional assumption of normality was assessed using graphical methods.

**Table 1:** Definition and classification of ANC adequacy

Indicators	Adequate use	Inadequate use
Number of ANC visits	At least three visits	Less than three visits
Time for fist visit	During first trimester	After first trimester
Content of ANC		
Core services (measurement of weight and height; assessment of blood pressure; fetal examination*; urine test; tetanus vaccination; health consultation)	Use of all core services at least once	No use of at least one of six core services
Optional services (*vaginal examination; blood test; iron/folate supplement; malaria prevention; ultrasound scan*)	Yes or No	Yes or No

* Including fundal height, abdominal circumference and heart rate measurement

### Ethical considerations

The establishment of the two HDSS was discussed with the local authorities and was approved by the Scientific and Ethical Committee of Hanoi Medical University and Ministry of Health of Vietnam. The participants were informed about the purpose of the project, their right to decline participation and to withdraw at any stage of the study. Verbal consent was obtained from all pregnant women. Data was analyzed and presented anonymously. It is considered that the mother's integrity has been affected to a minimal extent. Mothers can receive advice from obstetric experts within the project for the problems they have during pregnancy or in the use of antenatal care. A small gift worth 30,000 VND is offered to each newborn baby.

## Results

### Women characteristics

According to table 2 almost all women were married, most women were 20-34 years old and nullipara accounted for around a half. The women in the rural setting were younger and had more births than those in the urban. Nearly 95% of the urban women had graduated at least from high school, while most rural women had a secondary school education or less. The dominant occupations were office staff in urban and farmer in rural areas. Only 10.4% of the urban women and 4.4% of the rural women were the head of household. The percentages of high risk pregnancy were 11.3% and 13.4%, respectively in the urban and the rural areas.

**Table 2:** Background characteristics of the women (%)

Variables	Urban (n = 814)	Rural (n = 1318)
**Age group**		
< 20	0.6	4.2
20-24	12.8	41.6
25-29	46.9	34.5
30-34	30.0	12.9
35+	9.7	6.8
Mean age of nullipara (95% CI)	26.7(26.4-27.0)	23.5(23.2-23.8)
**Education**		
Secondary or less	5.9	56.7
High school	31.8	26.6
Higher than high school	62.3	16.7
**Occupation**		
Farmers	2.5	64.4
Office staff	57.4	10.2
Workers	6.5	10.1
Business/services	20.3	10.5
Others	13.4	4.8
**Head of household**		
Yes	10.4	4.6
No	89.6	95.4
**Marital status**		
Married	99.1	98.1
Unmarried	0.9	1.9
**Parity**		
1	56.5	46.6
2	39.1	35.4
3+	4.4	18.1
**High risk of pregnancy**		
Yes	11.3	13.4
No	88.7	86.7

### Number of ANC visits

Close to 100% of all pregnant women in the two sites had at least one ANC visit during pregnancy. However, the average number of visits as well as the proportion of women who received three or more visits was significantly higher in urban than in rural areas. There was a statistically significant difference between nullipara and multipara in the rural area for both the average number of visits and the percentage of women using three or more visits (table 3).

**Table 3:** Frequency of ANC visits, by parity (%)

Number of visits	Urban			Rural		
	
	Nullipara (n = 460)	Multipara (n = 354)	Total (n = 814)	Nullipara (n = 614)	Multipara (n = 704)	Total (n = 1318)
0	0.4	0	0.3	2.0	3.8	3.0
1	0	0.6	0.3	5.1	8.8	7.1
2	2.0	2.8	2.3	10.4	14.9	12.8
3+	97.6	96.6	97.2	82.6	72.4	77.2
Mean* (95%CI)	7.9 (7.6-8.2)	7.4 (7.0-7.7)	7.7 (7.5-7.9)	4.8 (4.6-5.0)	4.0 (3.8-4.2)	4.4 (4.2-4.5)

*p < 0.001 for t-test for mean values between areas and within area.

### Contents of ANC

According to table 4 the urban women used much more services than those in the rural area. A large number of women in the rural area did not receive simple and essential services, such as physical measurement, blood pressure assessment, and urine test. The percentage of women who received health consultations during ANC visits among the urban women was 4.2 times than in the rural. There was no significant difference in contents of ANC between nullipara and multipara, in both settings.

**Table 4:** Content of ANC used by nullipara and multipara women (%)

	Urban			Rural		
	
ANC services	Nullipara (n = 460)	Multipara (n = 354)	Total (n = 814)	Nullipara (n = 614)	Multipara (n = 704)	Total (n = 1318)
**Core services**						
Fetal examination*	99.1	99.4	99.3	82.8	77.1	79.8
Body weight and height measurement*	98.3	98.9	98.5	58.7	56.7	57.6
Blood pressure assessment*	97.8	97.7	97.8	60.7	57.7	59.1
Urine test*	89.3	86.7	88.1	38.1	33.2	35.5
Tetanus vaccination	92.6	92.1	92.4	94.3	89.4	91.7
Health consultation*	87.0	81.6	84.6	24.9	22.3	23.6
***All core services***	*82.6*	*79.1*	*81.1*	*22.0*	*18.9*	*20.3*

**Optional services**						

Folic & Iron supplement*	96.7	94.9	96.0	87.5	73.3	80.0
Blood sample test*	74.8	72.0	73.6	22.4	17.9	20.0
Ultrasound scan*	99.6	99.4	99.5	98.5	95.3	96.8
Mean number of scans**	6.0 (n = 71)	6.0 (n = 46)	6.0 (n = 117)	4.0 (n = 106)	3.0 (n = 116)	3.5 (n = 222)

*p < 0.001 for Chi square test for urban - rural comparison

**p < 0.001 for t-test for comparison of mean values between the two areas

Almost all women in both sites received ultrasound examination during pregnancy. Ultrasound was also the ANC service that was used most by the rural women (96.8%). The number of ultrasound scans was not recorded at the beginning of the study, only for a subgroup of women recruited during the last three months of the study. It shows that women in the urban area used significantly more ultrasound scans than those in the rural, table 4.

### ANC adequacy

**Figure 2:**
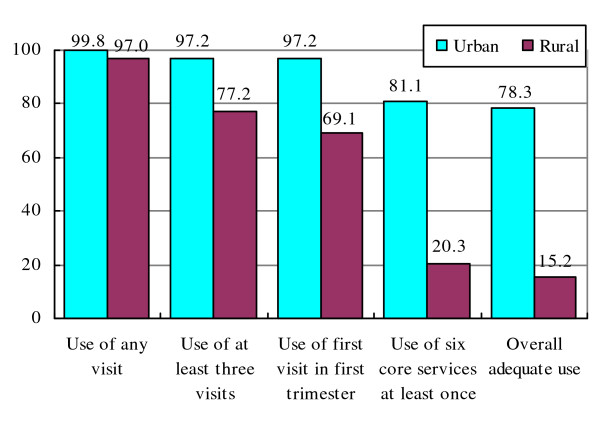
**Percentages of women who used ANC from different providers**.

Figure 2 shows the different level of ANC adequacy for the urban and the rural areas. The proportions of any ANC visit were almost the same in the two settings but urban women had their first ANC visit earlier and had more visits than rural women. The difference between urban and rural areas was still larger regarding the adequacy of utilization: the urban-rural ratio increased from 1.3 for the use of at least 3 visits (p < 0.05) and 1.4 for the attendance of ANC in the first trimester (p < 0.05) to 4.0 for the use of sufficient services (p < 0.001) and 5.2 for the adequate use (p < 0.001).

### Type of ANC utilization

For the rural women, the most common choices for ANC utilization were CHCs, private clinics and district hospitals. In the urban group, central or provincial hospitals were most commonly used. Most women in the two sites received ANC from the both public and private sector. The urban women used more public health facilities and fewer private for ANC than the women in the rural area. There was a statistically significant difference between nullipara and multipara regarding public/private type of ANC utilization in the urban area (table 5).

**Table 5:** Type of ANC utilization by nullipara and multipara women (%)

	Urban			Rural		
	
	Nullipara (n = 458)	Multipara (n = 354)	Total (n = 812)	Nullipara (n = 602)	Multipara (n = 677)	Total (n = 1279)
**Health facilities**						
CHC/Maternity*	7.4	10.2	8.6	69.3	67.5	68.3
District hospital*	7.0	5.7	6.4	58.1	56.1	57.1
Provincial hospital*	46.3	40.4	43.7	6.8	4.9	5.8
Central hospital*	72.3	68.6	70.7	2.5	3.4	3.0
Private clinic*	38.9	28.0	34.1	65.5	62.6	64.0
Private hospital	15.5	13.0	14.4	13.3	11.2	12.2
Others	0.7	0.8	0.7	1.5	0.9	1.2
**Public/private sector**						
Only public*	52.2	62.3	56.6	28.9	31.9	30.5
Only private*	5.0	4.8	4.9	8.7	9.6	9.2
Both*	42.8	32.9	38.5	62.4	58.5	60.4

*: p < 0.001 for Chi square test for rural - urban comparison

### ANC expenditure

The urban women spent 1.06 million VND (equal to 55 USD) for ANC utilization, 7.1 times the corresponding amount for the rural women. The women in the urban area paid 140.7 thousand VND per visit, 4.4 times compared to the rural women. The expenditure per visit at the private sector is higher than at the public sector in the urban area and conversely in the rural (table 6).

**Table 6:** Expenditure related to the use of ANC (mean, 95% CI; 1.000 VND)

Women used ANC at	Urban			Rural		
	
	n	Total expenditure	Expenditure per visit	n	Total expenditure	Expenditure per visit
Only public	459	968.0 (882.5-1053.4)	143.8 (131.6-156.0)	386	94.4 (66.9-121.9)	27.2 (20.4-34.0)
Only private	40	958.9 (700.0-1217.6)	122.8 (103.3-142.2)	117	137.3 (108.7-165.8)	40.1 (35.1-45.1)
Both public and private	312	1210.5 (1109.7-1311.2)	138.9 (129.1-148.6)	764	181.8 (168.7-194.8)	33.3 (31.5-35.1)
All women	811	1059.5 (995.8-1123.1)	140.7 (132.8-148.6)	1267	149.5 (137.6-161.4)	31.7 (29.3-34.1)

## Discussion

The percentage of women who used at least one ANC visit in this study is much higher compared to other low and middle income countries such as Lao PDR (59%) [24], India (73,9%) [25]. It is also higher than results from previous studies in Vietnam (71% in 1999; 50% in 1988) [21]. This indicator already reached the national target for 2010 [15]. The reasons for the increased use of ANC can be the socioeconomic development in Vietnam, especially in Hanoi, where the socioeconomic condition is better than in other provinces. It can also be due to achievements of the national programme for maternal and child health during the last 20 years [15]. However, for ANC to be effective in preventing adverse pregnancy outcomes, women should have adequate use with enough visits at appropriate times and sufficient services.

The result shows that urban women had more visits and used more services than women in the rural areas. This is in line with results from some studies in Nepal (2004) and Ecuador (2005) but in contrast to one study in Guatemala in 2002 [11]. Similar results were also found in some previous studies in Vietnam [16,20]. The urban area differs both qualitatively and quantitatively from the rural area in many ways, not only in the characteristics of women and their household but also in the general context of the two settings. All such differences at both individual and district levels may contribute to the difference in ANC utilization.

In this study, there were minor differences in the quantity of any ANC used between the two areas but the gap in the ANC adequacy was quite large. Two indicators, the mean number of visits and the percentage of women having at least three visits were used in almost all previous studies on ANC utilization in Vietnam. In some cases they showed disparity in the quantity of ANC visits between two settings. The urban-rural gap in percentage of women having three or more visits from our study was higher than in the survey conducted in 2002 (72.6% and 48.4%) [16]. It indicates a faster improvement in ANC coverage in urban than rural areas during the last decade. Although the average numbers of visits in both areas were higher than the national recommendation, a significant number of rural women still had fewer than three visits.

The difference between the two areas in terms of time for the first visit was even larger than the frequency of ANC. Early use of ANC plays an important role for the effect of the ANC programme and the national guidelines recommend that the first ANC visit should be made during the first trimester. However, this indicator is not frequently used for monitoring and evaluating ANC utilization in routine statistics at national level [14,15]. Previous studies in Vietnam also used different cut-off points to define a sufficiently early first ANC visit [18,20,21].

The late attendance in ANC among the rural women is possibly due to women's lack of awareness about the importance of early use of ANC among women. Another potential reason might be that the proportion of women with more than two children is higher in the rural area than in the urban. With the "1-2 children" policy, women who already have had more than two children might want to hide their pregnancy as long as possible, to avoid penalties. As a consequence, they may not attend ANC or start ANC visit later than the others [20,26].

Indicators such as frequency or initiation of ANC visits address mainly the quantity aspect of ANC utilization. In our opinion, the indicator of ANC content should show more completely the quality of ANC utilization. The proportion of women receiving all ANC might be underestimated due to "recall bias" in cross sectional surveys [21]. That may be one of the reasons why ANC content has not been used as one indicator of ANC adequacy in the national health survey or health statistics year book [14,27].

This study asked about all ANC services with six core services recommended in the national guideline for uncomplicated pregnant women. The results show that the use of ANC services was still poor, especially in the rural area, where less than one-fourth of the women used all six core services. A large number of the rural women did not receive simple and cost effective assessments such as weight, height and blood pressure measurement or urine test and then might miss the opportunity to detect early pregnancy conditions like pre-eclampsia [3]. Health education is one of the essential components of an ANC visit and the low proportion of women who received health consultations in this study indicates poor communication between health workers and women in the rural areas. The inadequate use of ANC service contributed mainly to the high proportion of overall inadequate use of ANC among the rural women (Figure 2).

In contrast to the inadequate use of core ANC services, almost all women had at least one ultrasound examination during pregnancy and ultrasound was offered in most ANC visits even though it is not a core content of ANC according to the national recommendations. The average number of scans is much higher compared to 2.7 scans in USA [28] and 3.2 scans in Canada [29] but it is still lower than in a previous survey in Hanoi in 2006 (6.3 scans and 8.3 ANC visits) [30]. The result indicates overuse of ultrasound examination possibly due to the commercialization of the Vietnamese health care system. Women, as well as health workers pay more attention to high technology services than to the core services. And it may be one of reasons for the inadequate use of ANC services among women. The appropriate use of ultrasound during pregnancy should be recommended in the national guidelines.

Some complex indices for ANC adequacy have been developed but they are mostly suitable for high income countries where high cut-off points of visits were recommended [21]. Combining indicators of quantity and content of ANC utilization should give more complete information on ANC adequacy. Using the most complex indicator defined earlier shows the largest discrepancy between the two settings. The large gap in ANC adequacy is mainly due to the inadequate use of ANC services among the rural women (Figure 2). To improve adequacy as well as quality of ANC utilization, the ANC content should receive more attention, especially in the rural area.

A difference in ANC utilization between urban and rural women was further seen in the types of health facilities where they had their ANC visits. Generally, the women in the urban area used more ANC at high level health facilities than the rural women. This difference might be explained primarily by differences in the availability of health facilities between the two areas. Within the area of only 10 km^2^, there are a lot of health facilities with high quality located in the urban Dong Da district, including public and private providers of ANC. Due to easier accessibility, a majority of urban women used ANC at secondary level health facilities. The proportion of urban women who received ANC at central and provincial hospitals, respectively, is higher than required for the majority of women, for whom ANC should be provided at CHCs or district hospital. It contributes partly to the overload at Obstetrics and Gynaecology departments in provincial and central hospital in Hanoi, which should be used only for pregnancy at high-risk or with severe complications.

The geographical accessibility might explain the high proportion of rural women who received ANC at CHCs and district hospitals, which are their closest health facilities. From these health facilities, women can receive ANC services at a lower cost, or even free (for vaccination, for example). However, in this study, rural women also received ANC from private health facilities more often than the urban women. The high proportion of rural women who received ANC from the private sector, with higher expenditure per visit than in public sector (table 6), implies that ANC cost is not the main obstacle for ANC use as mentioned in one previous study [26]. Although using more ANC from the private sector, the rural women spent less money for ANC than urban women. It might be explained by the lower number of visits, and the poorer services they had during ANC visits.

The high proportion of women who used ANC at private health facilities can possibly explain the poor adequacy of ANC in the rural area while the compliance with national guidelines in the private health sector is still unclear [13]. On the other hand, the large number of women who received ANC at central hospitals is also a problem. Sometimes, women visiting these health facilities went just to check on the status of the fetus so health workers might consider them as ordinary patients and not strictly follow the ANC guidelines.

The study gives an overall picture about the ANC adequacy and shows the large gap between rural and urban area using a considerably large sample and several indicators. The same data collection and management methods were used in both areas. There are some technical differences between the two HDSS which are assumed to be of no importance for this study. Previous studies on the use of ANC in Vietnam were mainly designed as cross sectional surveys in women aged 15-49 or on women who had given birth already at a specific area [17,21,27]. Data was collected only one time after delivery and might be strongly influenced by "recall bias". This study used two cohort surveys to follow pregnant women, in which information was collected every three months to reduce "recall bias". Instead of using only one or two indicators, the study used a set of indicators to assess the adequacy of ANC utilization. So it is expected to give a more comprehensive picture about the disparities between rural and urban areas regarding ANC adequacy.

The results indicate the need to have new guidelines with separate requirements for urban and rural areas, detailed instructions for specific groups and clear recommendations on the appropriate use of ultrasound during pregnancy. Of course strictly monitoring the compliance to ANC guidelines among health workers at different levels would be an important solution to improve the ANC adequacy. A comprehensive list of indicators is necessary to monitor and evaluate the implementation of the ANC programme at national level.

The prospective assessment of ANC utilization might not be comparable to surveys using retrospective methods with possibly larger recall bias. The present results show improvement in ANC use by women compared the retrospective study in 1999 but this can be partly due to that estimates have different biases.

As the two sites are unique, the results cannot be generalized in a formal statistical sense. Their usefulness for other settings depends on their degree of similarity with the investigated sites in various aspects. The contexts of DodaLab and FilaBavi are not representative for the whole country. Hanoi generally can be expected to have one of the best situations also in its urban parts Vietnam. However, the disparities between the urban and rural areas within Hanoi do suggest the general existence of urban - rural gaps in ANC adequacy.

## Conclusions

The study reveals a large disparity between the rural and urban area in the use of antenatal care. Rural women attend ANC later, had fewer visits and used much fewer services than urban women. They use more ANC at the private sector and at the primary level of public health sector than women in the urban area. Despite the inadequate use of essential ANC services, women in both areas use more ultrasound examination than would be necessary.

The large disparity in ANC adequacy between the rural and urban site within one province implies a large gap between regions in Vietnam and indicates a need to give more priority to the rural and remote areas. It also suggests a revision of the national strategy with more detailed guidelines and indicators for evaluation. For improved effectiveness, the ANC programme should pay more attention to the content of care, rather than number of visits.

## Competing interests

The authors declare that they have no competing interests

## Authors' contributions

TKT contributed to all aspects of the implementation. BE and MP were involved in the conception and design of the research idea, analyses and interpretation of the data and in revising the manuscript. NTKC and NDH were involved in designing the study, coordination and acquisition of the data and in revising the manuscript. KG, GR and HR were involved in the conception of the study, in drafting and revising the manuscript. All authors read and approved the final manuscript.

## Pre-publication history

The pre-publication history for this paper can be accessed here:

http://www.biomedcentral.com/1472-6963/11/120/prepub
